# Teaching Reform to the Biology Major During the COVID-19 Pandemic: A Study of the Method of Teaching Industrial Innovation and Entrepreneurial Talents

**DOI:** 10.3389/fpsyg.2022.843485

**Published:** 2022-05-09

**Authors:** Zhe Liu, Jingwei Wang, Zhiming Liang, Hongbo An, Liyang Li, Zhongjing Zang, Jing Li, Yang Xi, Tong Han, Shaobin Liu, Cheng-Hao Jin

**Affiliations:** ^1^College of Life Sciences and Biotechnology, Heilongjiang Bayi Agricultural University, Daqing, China; ^2^National Coarse Cereals Engineering Research Center, Daqing, China; ^3^College of Science, Heilongjiang Bayi Agricultural University, Daqing, China; ^4^Department of Food Science and Engineering, College of Food Science and Technology, Heilongjiang Bayi Agricultural University, Daqing, China

**Keywords:** innovation, entrepreneurship, training, biology major, cultivating talents

## Abstract

The biology major has developed rapidly in recent years. Biology is a science that penetrates every aspect of human life and is one of the core majors in most agricultural colleges and universities. However, many teachers lack practical experience in the subject. To overcome this problem, in recent years, we have been trying to introduce new reforms into our teaching. This article provides some insight into the way that biology majors have been reformed, which will help educators in agricultural colleges and universities. At present, teachers implement the “Industrial Innovation and Entrepreneurship Talent Cultivation” (IIETC) model, but it is not clear whether this helps biology majors to master the course and improve their practical skills. In this study, the IIETC model is outlined, and the academic achievement and satisfaction of students taught under the IIETC model are assessed. A *T*-test is used to examine potential differences between IIETC and traditional teaching models. In-depth interviews and questionnaires were given to two groups of students who followed different teaching models as part of an exploratory study. The aim was to explore how effective IIETC is at helping biology majors master the course and improve students’ wellbeing. Our results show that compared with traditional teaching methods, the IIETC model has a significant positive impact on the academic performance and happiness of biology students. Students trained under the IIETC model were more active and scored more highly in their final exams. They were more likely to feel that they had achieved success and happiness through the course (*P* = 0.03). The outcomes of this research reveal a novel teaching reform that improved students’ enthusiasm for innovation and entrepreneurship during the ongoing COVID-19 pandemic. The effects are very encouraging and deserve further exploration and expansion in future work.

## Introduction

Under the background of the COVID-19 pandemic, the endowing status of biology talents by employers has put forward a severe test for the employment of biology college students who are about to graduate. Facing the stressful employment situation, more and more college students realize that only with innovative ideas and entrepreneurial thinking can they be competitive in the increasingly fierce competition and rapidly changing world (Katper et al., 2020; Afshan et al., 2021; Liu et al., 2021; Tunio et al., 2021a,c). To improve the quality of teaching during the COVID-19 pandemic, teachers have implemented the “Industrial Innovation and Entrepreneurship Talent Cultivation” (IIETC) teaching method. Industrial innovation and entrepreneurship are reflected through cooperation between schools and enterprises (Hu et al., 2021). Students have two mentors: one is a teacher in school, and the other is in an enterprise. Entrepreneurship education has advantages for cultivating talents and developing practical skills (Fan, 2021).

Entrepreneurship education began in 1947 when Myles Mace offered the course “start-up business management” for MBA students of Harvard Business School. This course is considered to be the starting point of entrepreneurship education (Liu et al., 2021). Entrepreneurship education is to train students’ entrepreneurial spirit, to train students from job seekers to job creators, to provide jobs, and create jobs (Liu et al., 2021). Our teachers provide entrepreneurship education for students majoring in biology and take responsibility for their practical skills (Chen et al., 2020). In recent years, teachers have faced increasing pressure to devise teaching methods that include modern educational innovations alongside core scientific knowledge, helping students to meet the expectations of employers and other stakeholders (Guimarães et al., 2017). In the mid-1990s, there was a gradual international movement toward dual eligibility, but this has slowed or stopped in the past decade (Khalil et al., 2018). China’s recent strategic guidance proposes to encourage undergraduate universities to become more focused on practical training. There are a lot of problems with how biology is taught. As a lot of enterprises’ data is confidential, it is difficult for teachers to obtain real and useful business case studies. Most teachers completely rely on courseware, teaching means and methods are single, unable to vividly interpret the nature of biology major.

Given the opportunities for educational development provided by COVID-19, teachers should adopt the IIETC model (Galindo-Martín et al., 2021; Ratten and Jones, 2021). The COVID-19 outbreak has forced students to stay in their hometowns instead of going back to school or to take online classes while schools are closed. The pandemic has transformed traditional offline education into online education (Dzara et al., 2019; An and Xu, 2021; Krouska et al., 2021; Troussas et al., 2021). IIETT teaching is part of the plan for education reform for students of a certain characteristics of the times. Our teaching methods are both online and offline, combining theory with practical experience of business (Geng et al., 2021; Hu et al., 2021). Teachers should strategically use the Internet and mobile devices to organize students, manage resources, and carry out tasks (Kanetaki et al., 2021; Yao et al., 2021). The outbreak of COVID-19 has forced teachers to switch freely between online and offline teaching methods. With the help of platform resources such as XuetangX provided by Tsinghua University and Superstar Learning, multiple teaching evaluation systems have been innovated and reformed (Ma et al., 2020; McCullough et al., 2020). In terms of content, teachers must make timely adjustments to their teaching methods in accordance with the latest industry trends (Tunio et al., 2021b). They must constantly update their content and teaching methods according to the market demand for relevant content and learning technologies. Once the risk of an outbreak in a particular region lowers from high or medium to no risk, students can enter enterprises and receive practical training from the mentors in the enterprise.

Corporate mentors, who are different from students’ tutors for their senior thesis, provide one-to-one mentoring for students. Graduates then rotate between different companies and attend standard training sessions. School teachers provide theoretical guidance on technical and research issues. Corporate mentors do the same, but they take a personal interest in the mentees’ professional (and sometimes even their personal) development (Li et al., 2020). Meanwhile, teachers can actively contact outstanding experts in relevant industries, who are the technical backbone of certain companies. They can invite these masters, who have rich practical experience, to enter the school and provide practical guidance. They can help students to solve the problems they encounter in actual workplace scenarios.

**Figure 1:**
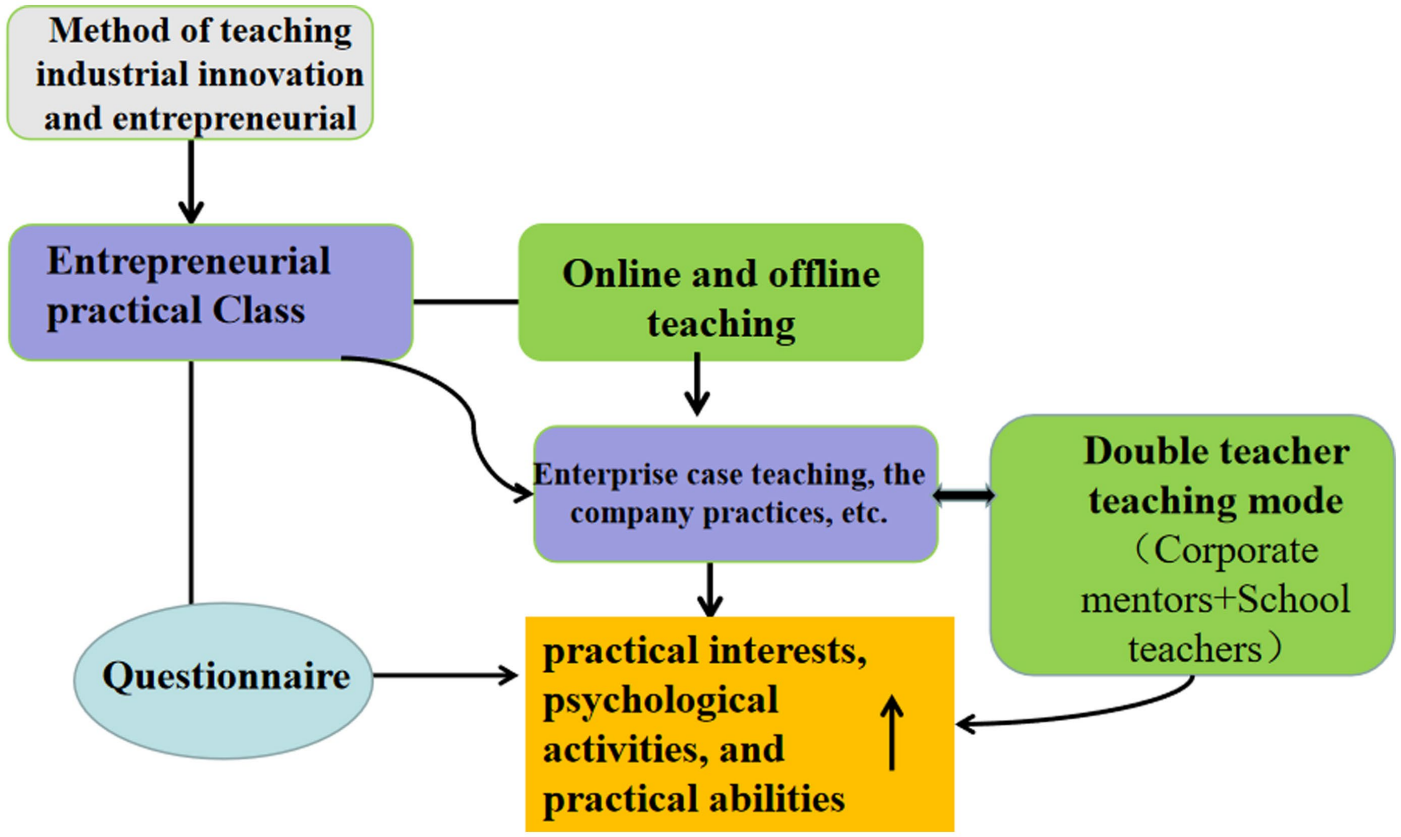
The relationship between the entrepreneurial interest of biology major students in and college teaching.

Moreover, curricular innovations require that attention is also paid to topics like the formation of professional identity, professionalism, and commitment to social accountability (Rogan and Anderson, 2011). Since experimental learning through practice is not ideal, students do not like to think. This means that there will inevitably be reforms to teaching models. The practical training provided by IIETC has offers a mature and effective model for innovation and transformation that has been very successful. This study was inspired by the expanding of innovation and entrepreneurship education during the COVID-19 pandemic. Since the implementation of the IIETC model, the relevance and effectiveness of teaching have been significantly improved, and the appeal of teaching has increased. The satisfaction rate of students majoring in biology has been rising year on year, as shown in Figure 1.

Autonomy and independence have always been core parts of student entrepreneurship (Rouleau et al., 2019). Therefore, we have established a set of practical teaching systems that cover a variety of technologies. In practice, we are constantly developing new, personalized courses. Also, we have set up a cloud classroom teaching platform. Online and offline teaching methods complement each other (Kang and Kim, 2021). We came up with the following hypothesis: if schools engaged in national practical education increase along with the growth of biopharmaceutical companies (and the sustainable development of biopharmaceutical companies is crucial), then the system can help schools to cope with future pandemics. We are currently assessing the IIETC model to check whether it allows biology majors to master their courses. We are trying to understand what the impact of improving practical ability is. Therefore, this study evaluates students’ academic performance and satisfaction under the IIETC model.

## Materials and Methods

### Sample of Students

In China, most students at the College of Life Sciences study professional course in their third year. The baseline pairs before the study were IIETC and traditional teaching type (TTT) students from Heilongjiang Bayi Agricultural University, as shown in Table 1.

**Table 1:** Baseline comparison before the study.

Variables	IIETC	TTT	*P-*Value
Admit a mark	medium	medium	>0.05
English achievement	medium	medium	>0.05
Academic performance before the study	good	good	>0.05

IIETC, Industrial Innovation and Entrepreneurship Talent Cultivation; TTT, Traditional teaching type.

A total of 432 students from these two groups were divided based on the two teaching methods and whether they had completed their final exams at the end of 2021. There was no statistically significant difference between the two groups when it came to other course-related variables, such as admission scores in English and pre-study courses (*p* > 0.05).

### Questionnaire

In the context of COVID-19, the psychological state of college students majoring in biology needs in-depth research and analysis. This research should focus on two aspects: teaching activities and practical psychology. This will help students to establish positive and practical psychological coping mechanisms, while also improving their practical interest and practical skills. To understand the impact of IIETC teaching on students’ psychology and academic performance during the pandemic, we designed a questionnaire with 16 questions.

In addition to their typical course assessments, students were asked to fill out a questionnaire about the course over several months (Xin et al., 2020). From September to November 2021, the questions were sent to students electronically. The responses were anonymous (Yao et al., 2021). A total of 228 questionnaires were issued. The questionnaire was divided into two parts. The first part focused on basic information about the college students, including their gender, the age, hometown, religious belief, and major. The second part focused on assessing the students’ psychology and practical skills. Then, the Likert five scale is used as a measuring tool to calculate the score of the subject’s cognitive feelings quantitatively (Zhang and Song, 2021). A total of 220 valid questionnaires were collected, a recovery rate of 96.49%, meaning that the data could be analyzed.

### Comparison of Student Performance Under Different Teaching Methods

Class 1 uses a traditional teaching methods, whereas Class 2 uses online and offline teaching methods and IIETC. To compare the performance of the two groups of students under different teaching methods, through interviewing and mobile teaching software “Super Star Learning,” the following measures were assessed as: the number of students participating in the two different teaching methods, attendance rate, homework completion, in-class responses, post-class feedback, student quality, teacher satisfaction, and students’ perception of pain (headaches).

We used “Super Star Learning” to compare students’ performance under the two teaching methods. Evaluating student performance involved average time spent watching videos, and the average score of in-class activities, consulting literature, viewing to answer first, taking group competitions, engaging in group tasks, and performing classroom exercises. In addition, the teaching effect and the distribution of students’ scores in the two different teaching methods were analyzed.

### Statistics

Graphpad Prism version 8.0 (La Jolla, CA, United States) was used to analyze the data from the questionnaire. The data were denoted as mean ± standard deviation. Furthermore, the differences between the two groups were compared using a *t*-test (two tailed). A value of *p* less than 0.05 was considered statistically significant, and a value less than 0.01 was considered markedly significant.

## Results

### Analysis of the Results of the Questionnaire

**Figure 2:**
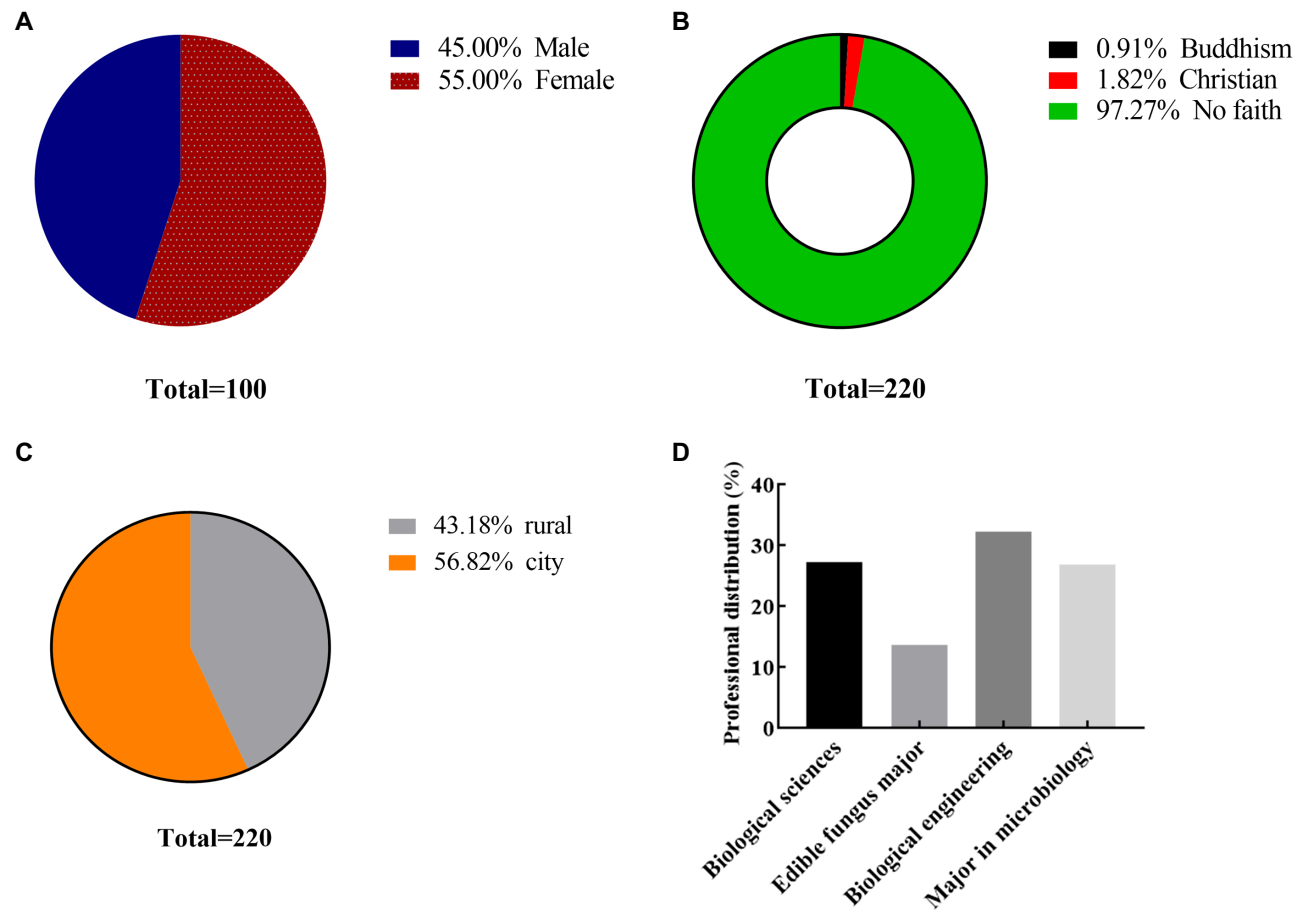
Basic information of the research object. **(A)** Gender ratio; **(B)** religious beliefs; **(C)** family situation; and **(D)** professional distribution.

**Figure 3:**
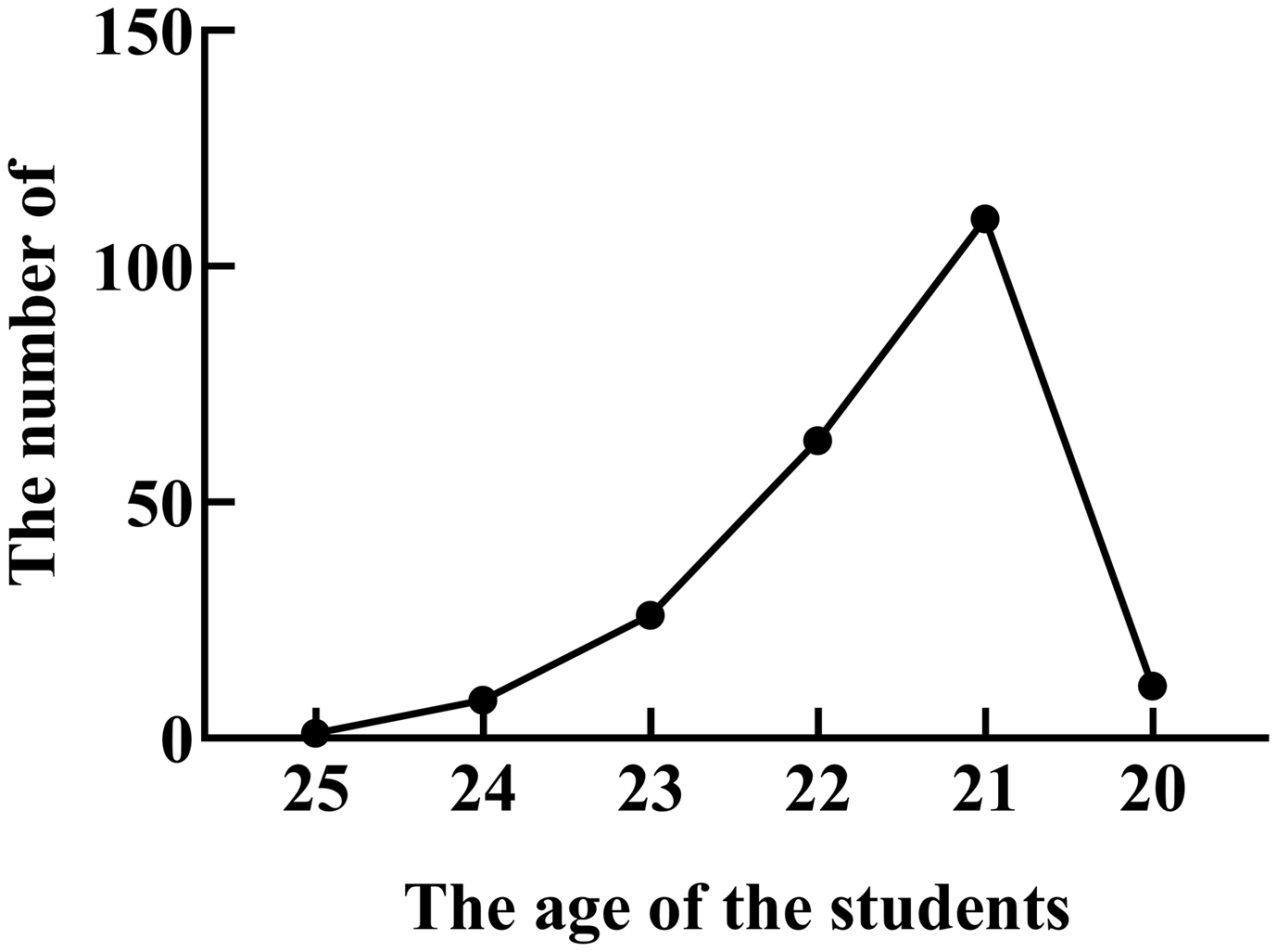
The age statistics of college students who participated in the questionnaire.

The questionnaire shows the basic information of college students majoring in biology, including their gender, family situation, and religious beliefs. As can be seen from Figure 2, the proportion of girls is 55.0% and that of boys is 45.0%. A 97.27% students have no religious belief, and the percentage of Buddhist and Christian students is less than 4%. A 56.82% of the subjects came from urban areas, and 43.18% came from rural areas. The number of male and female students in urban areas is the same as that in rural areas. These does not affect our statistical results. The students majored in biological sciences (27.2%), edible fungi (13.64%), microbiology (26.825), and bioengineering (32.27%). The results of the questionnaire show that biological engineering students paid more attention to developing their practical skills. The age statistics of college students who participated in the questionnaire were shown in Figure 3. As can be seen from Figure 3, the age range of students participating in the questionnaire ranges from 20 to 25 years old, among which the most students are 21 years old.

**Figure 4:**
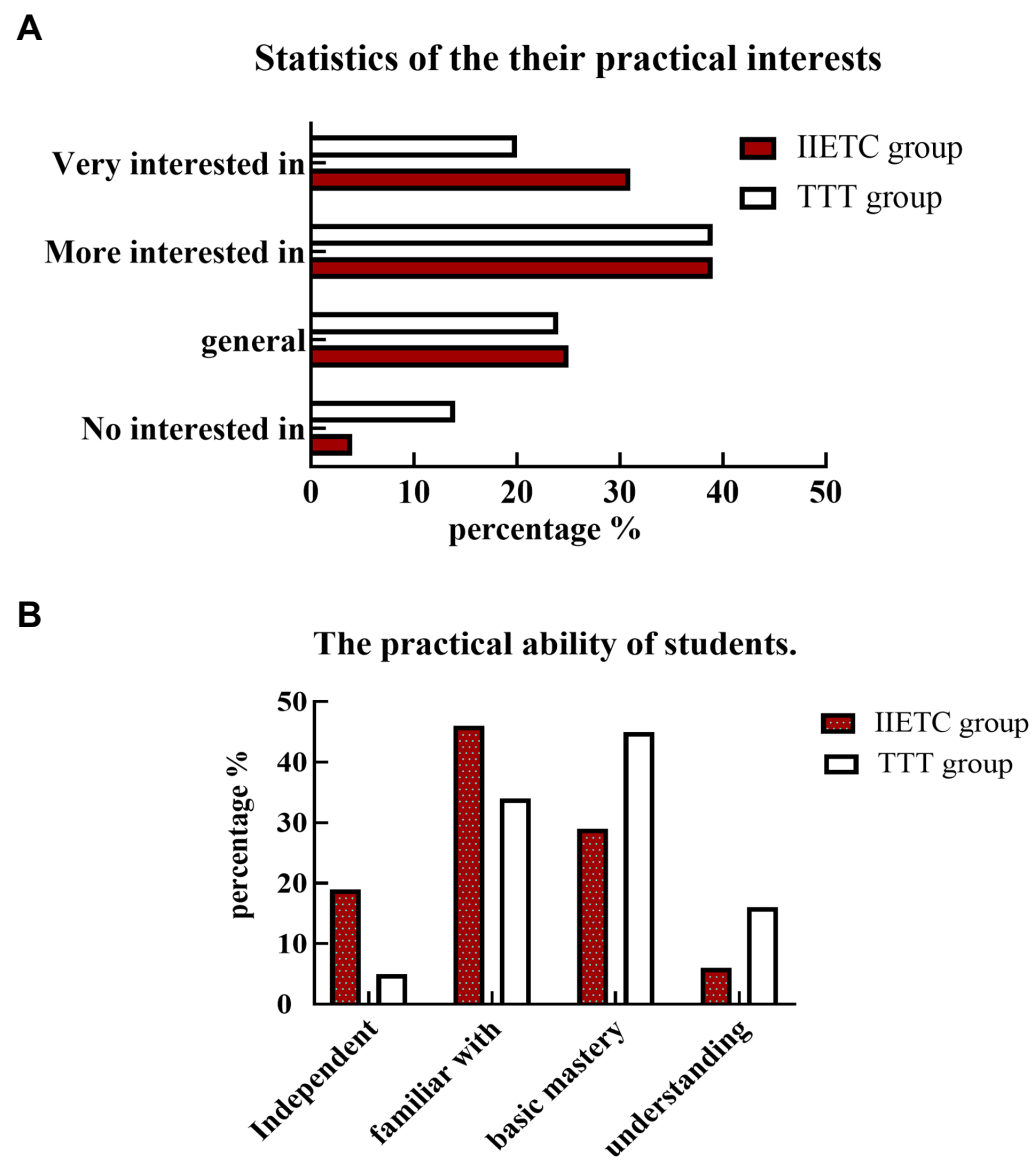
Statistics on students’ psychological activities and practical ability. **(A)** Statistics on their practice interest; **(B)** statistics on students’ practical abilities.

To study the influence of the IIETC teaching method on the practical psychology of college students majoring in biology, we conducted a statistical analysis of the questionnaire survey. We analyzed the students’ practical interests, psychological activities, and practical abilities. The results are shown in Figure 4. It can be seen from Figure 4A that 31.09% of the IIETC students were very interested in practical skills, 25.21% were moderately interested in practical skills, and 4.20% were not at all interested in practical skills. A 20.79% of TTT students were very interested in practical skills, 24.75% were moderately interested in practical skills, and 14.85% were not at all interested in practical skills. Compared with the TTT group, the percentage of students in the IIETC group who were very interested was significantly larger (*p* < 0.01); the percentage of students who were not interested at all was significantly smaller (*p* < 0.01). There was no significant difference in the percentage of those who showed some interest in practice (*p* > 0.05). The investigation and analysis of students’ practice psychology show that students have good practice enthusiasm under the new teaching method environment (Reis, 2018).

In THE IIETC group, 6% of students had no practical skills at all, 19% of students had independent practical skills, and 46% of students were familiar with practical skills. In the TTT group, 16% of students had no practical skills, 5% of students had independent practical skills, and 34% of students were familiar with practical skills. Compared with the TTT group, the IIETC group had significantly better practical skills and were more familiar with practical skills (*p* < 0.01, Figure 4B); the IIETC group had a significantly lower percentage of students who had a basic grasp of practical skills and who understood practical skills (*p* < 0.01).

### IIETC Has a Significant Positive Impact on Student Welfare

This study investigated the impact of IIETC teaching methods on student wellbeing. To our surprise, the students in the IIETC group all completed the non-essential study module assignments. We found that biology students in the IIETC group showed higher levels of cohesion on campus and had significantly reduced levels of anxiety, stress, and depressive symptoms. The innovative IIETC model helps to limit the pressures of the course, benefitting students’ mental health. Students in the IIETC group reported less “pain” (defined as depression, physical discomfort, hostility, or anxiety) on the questionnaire than students in the TTT group (see Table 2).

**Table 2:** The impact of IIETC on the students’ performance of biology major.

Behavioral engagement indicator	IIETC	TTT	*P-*value
Registration number (*n*)	236	196	0.04
Attendance	100%	98.50%	0.6
Assignments completed	All	78(39.8%)	0.04
Response (In-class group)	66(28%) students voluntarily posted responses	25(12.8%) prompted interactions during class	0.01
Feedback (Out-of-class group)	44(18.6%)	7(3.6%)	0.01
Quality of student	Substantive and constructive	Superficial	
Perceived pain (Headache)	0	5	0.02
Faculty satisfaction	High	Low	0.03
perceived pain (headache)	0	5	0.02

n, No. of students; IIETC, Industrial Innovation and Entrepreneurship Talent Cultivation; TTT, Traditional Teaching Type.

### Comparison of Student Performance Under Different Teaching Methods

This study describes the observed effects of IIETC on the teaching activities of a single biology course. Table 3 summarizes the assessment of the classroom performance of the two classes. We were surprised to see differences in the quantity and quality of excellent results between the two groups.

**Table 3:** The impact of IIETC on students response (in-class group).

Variables	IIETC	TTT	*P-*value
Average time spent watching videos (Minutes)	43.19	34.22	>0.05
Average score for in-class activities	30.1	16.4	<0.01
Consulting literature	82.10%	56.30%	<0.01
Viewing to answer first	25.00%	9.80%	<0.01
Group competition	66.10%	12.60%	<0.01
Engaging in group tasks	98.52%	74.34%	<0.05
Practice in class	84.70%	42.30%	<0.01

From an analysis of the mobile teaching software “Super Star Learning,” there was no significant difference between the TTT group and the IIETC group when it came to the average duration of teaching that involved watching a video (*p* > 0.05). The average score for classroom activities, literature reviews, quick answers, animal cell engineering group competition, group task completion rate, and classroom exercise results was significantly improved in the IIETC group (*p* < 0.01). A high completion rate for group tasks demonstrates teamwork. In other words, biology students trained using the IIETC method have high levels of cohesion on campus (Zhao et al., 2020).

**Figure 5:**
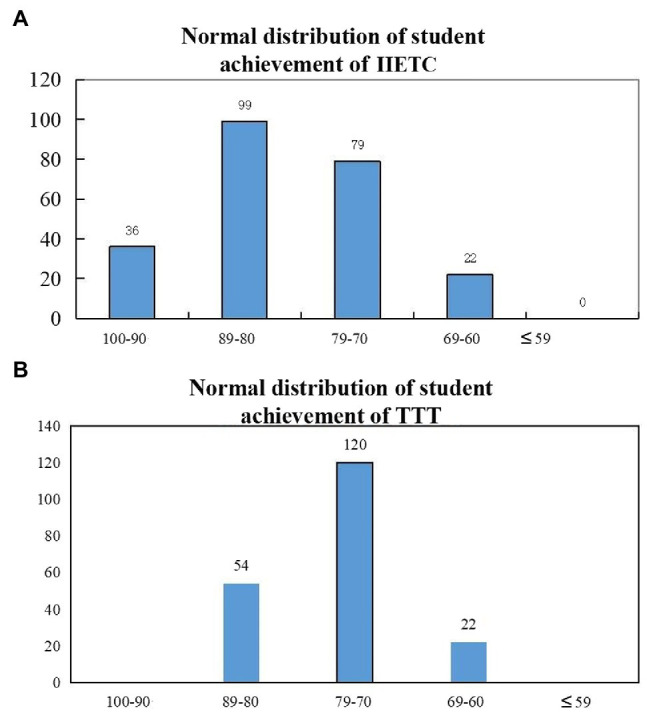
Normal distribution of student achievement. **(A)** IIETC culture conditions; **(B)** TTT culture conditions.

In the IIETC group, the effect of the teaching was positive. The teachers were satisfied with the results. Most students could analyze and solve problems reasonably. The percentage of students who achieved excellent was 15.25%, and the average score in the class was 84.52%. In the TTT group, teachers were generally satisfied with their grades, and the average score in the class was 77.01%, though there were no excellent grades and no low grades (Table 4). The normal distribution of the two scores was reasonable, as shown in Figure 5.

**Table 4:** The achievement analysis of biological students with two culture conditions.

	Analysis of results of group IIETC	Analysis of results of group TTT
Lack of the number of test	0 (0%)	0 (0%)
100–90 score	19 (15.25%)	0 (0%)
89–80 score	99 (41.94%)	54 (27.55%)
79–70 score	79 (33.47%)	120 (61.22%)
69–60 score	22 (9.32%)	22 (11.22%)
≤59	0 (0%)	0 (0%)
Average score (the total score was 100)	86.31	77.01
Overall number of people	236	196

## Discussion

The purpose of this study was to explore the current situation and suggest reforms to the education of students in biology, particularly given the situation of the pandemic. The purpose was also to support the development of the biological industry through the cultivation of strong talents. Biology undergraduates lack practical skills when they graduate because most of them do not have the opportunity to work with businesses while at school. They do not understand how to pursue a career after graduation. Moreover, many students change their careers to be in sales (Wang et al., 2021b). This is not a minority phenomenon. It may be because schools do not stimulate students’ interest in biology as a major or because many students are confused about how to plan their future careers (Kong et al., 2020). Therefore, we have compared teaching methods focused on innovation and entrepreneurship with TTT. The IIETC method can increase students’ enthusiasm for professional development and fulfillment. It can even limit their pain and increase their happiness. By working with schoolteachers and business mentors, students can discuss any problems they have, allowing them to strengthen their relationship with their teachers and to identify and resolve any physical or mental health problems during the pandemic. Innovation and entrepreneurship among technical professionals are important for the future development of the economy and society.

During the ongoing COVID-19 pandemic, teachers should consider the mental health of students, as well as the practical impact of their teaching methods (Dong et al., 2019; Wang et al., 2021a). Teachers can use interactions with students to promote and consolidate their online, independent learning. IIETC is a new method of education. By organically integrating online and offline education with cooperation between schools and businesses, it can help students to increase their knowledge and strengthen their theoretical understanding through comprehensive learning (Xi and Liu, 2020). Students taught using the IIETC method seem to be more active in curricular learning and are more capable of solving real problems and have high enthusiasm for innovation and entrepreneurship. This gives them a deeper understanding (Wei, 2021). Since the IIETC reform combines theory and practice, it allows students to develop their thinking and grow. It combines individual unity and overall development. There is a great difference between theoretical education and practical life. The practical skills of college students will arouse students’ thinking. The IIETC method, the double teacher teaching mode, improves the ability of team cooperation and project communication needed by the talents to start their business. We should pay close attention to the relationship between students and IIETC strategies to better understand how IIETC strategies affect students’ interest in academic research. We should help teachers to promote student development. Teachers could increase their research, learn from successful experiences at home and abroad, and try to understand and respect the opinions of different industries and universities. They should increase their cooperation with enterprises and promote enthusiasm in both sectors. In this study, teachers improved the teaching effect and provided the most solid guarantee for the establishment of an education platform and applied university. This study makes practical contributions to the cultivation of students’ innovation and entrepreneurial skills.

## Conclusion

The present study shows that IIETC teaching is an effective teaching method and is appreciated by biology students. It deserves to be introduced as a teaching method in other subjects. These effects are very encouraging and deserve further exploration and expansion in future work in order to determine the sustainability of the methods proposed in this study.

Limitations of this work include that it focuses on biology major students as well as it assessed the utilization of IIETC but not compared to Learning Management Systems approaches. This research serves for recommending teachers to enrich the tutoring process by using alternative innovative approaches with pedagogical potential as well as students to be obtained their practical ability.

## Data Availability Statement

The original contributions presented in the study are included in the article/supplementary materials, further inquiries can be directed to the corresponding author.

## Author Contributions

ZL conceptualized the manuscript and provided data and developed Figure 1 and Tables 3 and 4. JW, ZL, JL, YX, TH, and SL conducted the statistical analyses. ZL and HA generated Figure 2 and Figure 4. LL and ZZ generated Figure 3 and Tables 1 and 2. CJ revised the paper. All authors contributed to the article and approved the submitted version.

## Funding

This work was supported by grants from the Higher Education Teaching Reform research undergraduate project of Heilongjiang Education Department (SJGY20210623 and SJGY20210651); Higher Education Teaching Reform research undergraduate project of Heilongjiang Education Department (SJGY20190484); Heilongjiang Province education science 13th Five-Year Plan 2017 annual record project (GBC1317101).

## Conflict of Interest

The authors declare that the research was conducted in the absence of any commercial or financial relationships that could be construed as a potential conflict of interest.

## Publisher’s Note

All claims expressed in this article are solely those of the authors and do not necessarily represent those of their affiliated organizations, or those of the publisher, the editors and the reviewers. Any product that may be evaluated in this article, or claim that may be made by its manufacturer, is not guaranteed or endorsed by the publisher.
